# Decentralization of birth registration to Local Government in Tanzania: the association with completeness of birth registration and certification

**DOI:** 10.1080/16549716.2020.1831795

**Published:** 2020-10-26

**Authors:** Christopher Sanga, Gregory Kabadi, Emilian Karugendo, Don de Savigny, Daniel Cobos Muñoz, Tim Adair

**Affiliations:** a Planning Monitoring and Evaluation Section, Registration Insolvency and Trusteeship Agency (RITA), Dar es Salaam, Tanzania; b Project Management Department, Tawi Consult Ltd, Dar es Salaam, Tanzania; c Statistical Methods, Standard and Coordination National Bureau of Statistics (NBS), Dar es Salaam, Tanzania; d Department of Epidemiology and Public Health, Swiss Tropical and Public Health Institute, Basel, Switzerland; e University of Basel, Basel, Switzerland; f Melbourne School of Population and Global Health, The University of Melbourne, Carlton, Australia

**Keywords:** Civil registration, vital statistics, Tanzania, birth registration, decentralization

## Abstract

**Background:**

In Tanzania only an estimated one-quarter of births are registered and certified. Birth registration uses a centralized system with geographic and cost barriers for families. A pilot decentralized birth registration system has been trialled in 11 of 26 regions, substantially increasing registration points, and enabling notification, registration and certification to occur in one step.

**Objective:**

This study compares completeness of birth registration and certification and achievement of key birth registration milestones in two districts where the birth registration system decentralized and two districts with the existing centralized system.

**Methods:**

Registration, notification, census and survey data were used to estimate birth registration completeness and quantify achievement of key registration milestones for births in 2012–16. These were compared between districts of Mbozi (decentralized in 2013) and Iringa (decentralized in 2016) and districts of Dodoma and Kibaha which remained centralized.

**Results:**

For births that occurred from 2012 to 2016, completeness of birth registration/certification (by early 2017) was higher in districts that decentralized (Iringa 60%; Mbozi 52%) than remained centralized (Kibaha 36%; Dodoma 20%). Introduction of the decentralized system saw completeness for births registered within 12 months of occurrence increase in Iringa from 1% in 2014 to 67% in 2016, and in Mbozi from 15% in 2012 to 36% in 2013 before falling and subsequently increasing to 53% in 2016. In contrast, completeness in centralized districts did not increase. Although a higher proportion of births are notified in centralized than decentralized districts, registration and certification occurs for all notified births in decentralized districts but only one-third in centralized districts.

**Conclusions:**

Benefits of a decentralized system are more proximate registration points and the merging of notification, registration and certification steps. The findings, while demonstrating the immediate impact of the decentralized system on completeness, also show that continued efforts are necessary to sustain these improvements.

## Background

Birth registration has many benefits for individuals, including to provide legal identity for citizenship and voting rights, as a requirement to access social security benefits and health and education services, and, more generally, as a fundamental human right [1–4]. At a societal level, complete birth registration within a national civil registration and vital statistics (CRVS) system is the best source of fertility statistics which are used to monitor birth rates and family planning programs, calculate early age and maternal mortality indicators, develop population projections and plan government service provision [1].

Complete birth registration by 2030 is the aim of Sustainable Development Goal 16.9, however in many countries, including Tanzania, birth registration is incomplete [5–7]. A review of CRVS system strengthening efforts found that effective interventions to improve birth registration have largely comprised some combination of supply (e.g. improved accessibility of registration), demand (e.g. awareness campaigns) and incentive (e.g. policies to encourage registration) components [8]. In Ethiopia, some factors that increased birth registration included registration facilities that charged lower fees for birth certificates, had shorter waiting times and more proximate to residents [9]. There is however a lack of empirical peer-reviewed studies on this topic, and some interventions, particularly related to incentives, are not necessarily generalisable to other populations.

**Figure 1.**
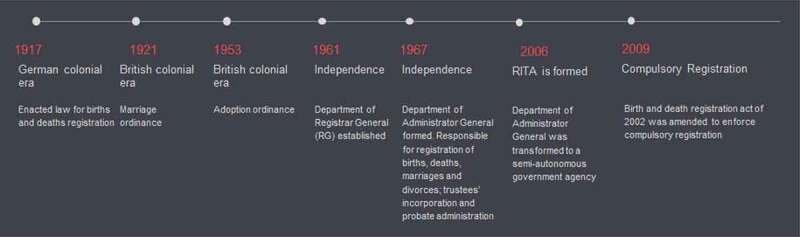
Timeline of development of the Tanzanian CRVS system

In Tanzania, civil registration has existed for over 100 years, but only relatively recently has compulsory birth and death registration been enforced [10]. German and then British colonial authorities maintained a birth and death register, but registration was not compulsory for native Africans (Figure 1) [10]. Over time, the Department of Registrar General, Department of Administrator General and then, since 2006, the semi-autonomous government Registration Insolvency and Trusteeship Agency (RITA) under the Ministry of Constitutional and Legal Affairs, have had responsibility for registration of vital events. Only in 2002 was the birth and death registration act amended to enforce compulsory birth and death registration for all citizens.

**Figure 2.**
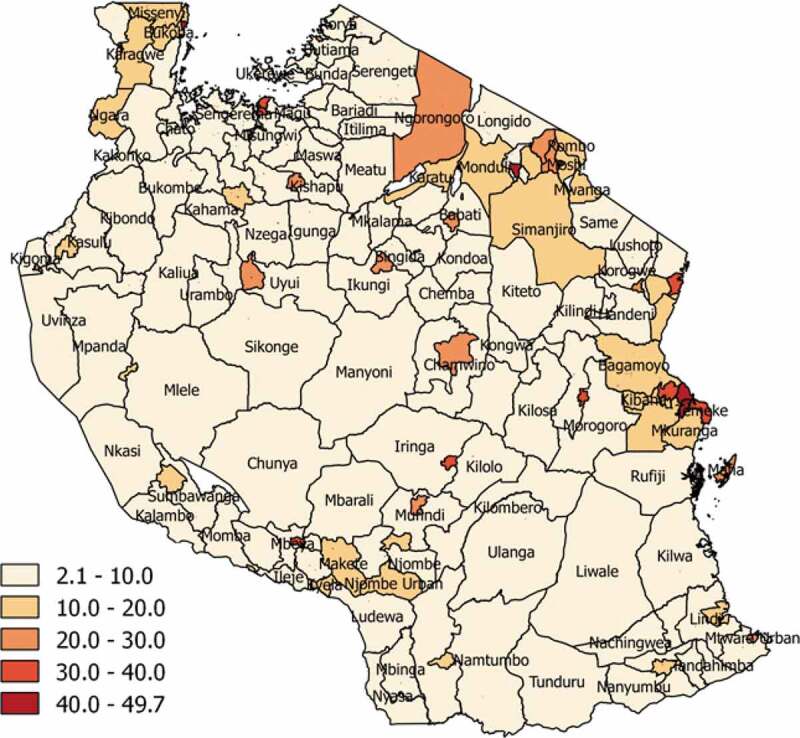
Birth registration coverage (% of population of all ages with birth certificate), by district, Tanzania Population and Housing Census, 2012

Tanzania’s completeness of birth registration is low, and the CRVS system cannot reliably be used to produce vital statistics. Just 24% of the UN-estimated 2,052,000 births in the country in 2017 were registered and certified [11]. Hence, fertility statistics are derived from censuses and surveys. For censuses, which were first conducted in 1967 and most recently in 2012, data collected on household births in the previous 1 or 2 years are used with demographic methods to estimate the level and age pattern of fertility [12]. The Tanzania Demographic and Health Survey (DHS) periodically collects detailed parent-reported birth histories from which fertility statistics can be derived, along with parent-reported birth registration. The 2015–2016 DHS revealed that according to parental self-report, just 26% of children aged less than 5 years had their birth registered, and just 14% had a birth certificate. Parental self-reported birth registration ranged from 16% in rural areas to 50% in urban areas, and from 8% in the poorest wealth quintile to 65% in the richest quintile [13]. Responses in the 2012 Census showed that possession of a birth certificate (among all age groups) varied widely across districts and was lower in more remote areas (Figure 2) [12].

Many Tanzanian government functions have decentralized in recent decades. In general, the term decentralization refers to the transfer of authority and responsibility for public functions from the central government to subordinate or quasi-independent government organizations and/or the private sector [14]. Beginning in 1972, Tanzania adopted decentralization policies focused on decentralizing key authorities and functions of government from the national level to the periphery to improve community participation in decision making, as found in other countries [15]. Along with other civil service reforms in the 1990s, civil registration services were decentralized to the district level, where all births and deaths could be registered by the District Administrative Secretary [16]. However, this did not impact coverage, even after making birth registration compulsory in 2009, because birth certification rates according to the DHS and Census remained only 13% [12,13].

Based on a comprehensive CRVS Assessment in 2014, Tanzania mainland has developed a national CRVS Strategy for 2015/16 to 2020/21 (awaiting approval of the government), which aims to establish a fully functioning and complete CRVS system and address the challenges of the centralized birth registration system. However, decentralization of the birth registration process has been advanced by the government through RITA with the development of the Under-Five Birth Registration Initiative (U5BRI). The U5BRI has declared health facilities and the Ward Executive Offices (WEOs) as official registration points; made registration free for the user; and made the first copy of certificate provided free of cost immediately after the registration. The initiative was piloted in Temeke District in Dar es Salaam in 2012. The U5BRI is now implemented in 13 out of 26 regions and has increased registration points from 183 district offices to 4,817 registration centres, comprising 1,736 ward offices and 3,081 health facilities. The larger number of registration centres that are health facilities rather than ward offices reflects that, nationally, 63% of births occur in health facilities [13]. The initiative has reduced steps of registration from three to only one (a hand-written certificate is issued at the time of registration). This system also applies a digital solution towards registration of births whereby mobile phones are used for uploading birth registration data directly to a central database, likely completed registrations forms are scanned and later linked to the uploaded record for further validation. Overall, in 2017, 42% of districts (58 out of 139) were implementing a decentralized birth registration system. The plan is to decentralize all districts by 2022. Further details of the process of birth notification, registration and certification are described in the Methods section.

The introduction of the decentralized U5BRI can potentially reduce the barriers faced by many Tanzanians in registering births and improve registration completeness. This study compares the performance of the system in two districts where decentralized birth registration has been introduced with two other districts where the existing centralized system still operates, in terms of:

- Completeness of birth registration/certification, including within 12 months of the birth
- Quantification of key birth registration milestones, specifically notification and registration/certification

## Methods

### Study setting

**Figure 3.**
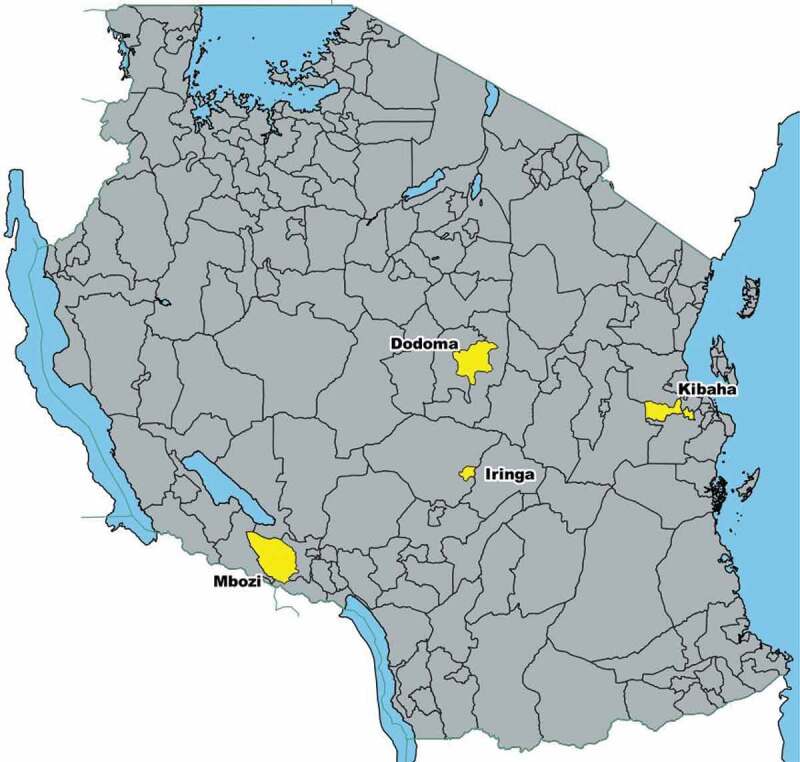
Location of study districts

This study was conducted in four districts of Tanzania: Mbozi in Songwe region, Iringa Urban in Iringa region, Dodoma in Dodoma region and Kibaha in Coast Region (Figure 3). Kibaha and Dodoma employ a centralized civil registration system and Iringa and Mbozi employ a decentralized civil registration system. The districts were chosen purposively. Iringa and Mbozi were chosen because these districts contain Sample Vital Registration with Verbal Autopsy (SAVVY) data, which had originally been planned to use in this study. Kibaha and Dodoma were chosen because they have a computerized birth registration system. According to the 2012 Population and Housing Census, the population of Kibaha was 198,697, Dodoma was 410,956, Iringa was 151,345 and Mbozi was 446,339. Kibaha has 81% of its population living in urban areas, Dodoma 52% and Iringa 100%, whereas Mbozi is more rural with only 17% of its population residing in urban areas.

### Study design

In the study, two districts (Dodoma and Kibaha) with a centralized birth registration system were analyzed and compared with two districts (Mbozi and Iringa Urban) with a system that decentralized during the study period. In Mbozi district the decentralized birth registration system was launched in 2013 and in Iringa it was introduced in 2016. In Mbozi and Iringa, upon introduction of the decentralized system, all children born from 2012 onwards were eligible to be registered.

In the centralized birth registration process in Tanzania, the system is mainly paper-based with only a few districts implementing a semi-digital system at the certification stage. While notification of births occurs at health facilities and, more rarely, ward offices, registration and certification occurs at the district headquarters. Therefore, parents or family members must travel to a district headquarters to register a birth and receive a birth certificate, often for long distances and at significant cost. This may be the explanation for variation of registration and certification coverage between large districts and small districts shown in Figure 2. In decentralized districts, registration occurs at several locations which are ward offices and health facilities within wards.

Measurement of achievement of milestones within a birth registration system, from the occurrence of the birth through to certification, is necessary to fully understand the system’s strengths and weaknesses. Past research has identified ten CRVS milestones that every CRVS system must fulfil to achieve its objectives; notification, validation and verification, registration, certification, sharing information, storage and archiving, compilation, quality control, generation and dissemination [17]. In this study, a case study approach was employed which compared, for both centralized and decentralized birth registration systems, estimated completeness of births for the first four CRVS milestones of: 1) notification; 2) validation 3) registration; and 4) certification, to measure missing birth data in each step of the process [17].

For purpose of this study the first four CRVS milestones were defined and quantified.

#### Notification

Notification is the capture and onward transmission of minimum essential information on the fact of birth or death by a designated agent or official of the CRVS system using a CRVS authorized notification form (paper or electronic) with that transmission of information being sufficient to support eventual registration and certification of the vital event [17]. In Tanzania’s centralized birth registration system, a birth is supposed to be notified within 90 days, otherwise it is considered as a late registration. An additional fee is charged for a birth certificate for a late registration, depending on the age of the child. In a decentralized system a notification, validation, registration and certification have been merged and the time to notify has been extended up to 59 months (before a child reaches age 5).

#### Validation

Validation is the act by which a relevant authority confirms that all necessary documentation about a vital event is correct. The registration process can then continue. It includes standard checks and procedures to ensure the information contained in the notification is correct [17].

In the centralized birth registration system validation is done once the notification forms reach the district registrar; further validation is also done when a parent visits the district registrar to finalize registration and obtain a birth certificate. In the decentralized system, validation is done at the time of registration and certification, however further validation might need to be done once records have been uploaded onto the database, and each uploaded record is further compared with a scanned image of the registration form.

#### Registration

Registration is defined as the act of formally registering an event at a civil registration office where details of the event are entered into the official civil register by the Registrar [17]. In the centralized birth registration system, once validation has been conducted, the forms are arranged in a systematic manner and registration is assumed to be completed, however, at this stage some of the details such as name of child and father’s name might be missing and are filled-in during certification stage. In the decentralized system a different approach is applied; the registrar cannot start completing the form unless he/she is satisfied that all necessary details are available, once the form is completed registration is also completed.

#### Certification

Certification is the issuance by the Civil Registrar of a legal document certifying a birth or death. This is usually in the form of a birth or death certificate [17]. In the centralized system certification is done by the district registrar, a type-written or computer printed certificate on a special paper is issued. In a decentralized system a hand-filled certificate is issued as soon after all necessary details for registering a birth have been completed, certification is done at a health facility or ward office which has been declared as a registration centre.

### Data

A range of data sources were used in the analysis: Numbers of registered and certified births for 2012 to 2016 from RITA, numbers of notified births for 2012 to 2016 from district registrars of Kibaha and Dodoma, population and birth data from the 2002 and 2012 Tanzania Census from Tanzania National Bureau of Statistics (NBS) and IPUMS-International, and regional percentages of deliveries in health facilities estimated by the 2015–16 Demographic and Health Survey [12,13,18,19].

### Completeness of birth registration/certification

Completeness of birth registration/certification was estimated as registered and certified births divided by the estimated true number of births. Separate databases exist for centralized districts and decentralized districts. Data were consolidated for the entire period from 1 January 2012 to 31 December 2016. We firstly measured completeness for all births that occurred from 2012 to 2016 and that were registered by early 2017. Even though Mbozi did not decentralize until 2013 and Iringa until 2016, it is expected the introduction of the decentralized system would improve completeness in earlier years because all children born from 2012 onwards were eligible to be registered. Data for centralized districts include all births which were certified as of 6 January 2017 whereas data for decentralized districts include all births which were certified and their records uploaded into the database as of 27 April 2017. This may partly explain low completeness rates in centralized districts in 2016. We also measured completeness for births registered and certified within 12 months from the date of birth, to assess the timeliness of registration and certification. All tabulations were made by mother’s usual residence.

To estimate the true number of births, 10% samples of the Tanzanian Census datasets for 2002 and 2012 were used. These samples were chosen systematically with sample weights provided and used in our analysis to calculate results weighted to the Census population. Data on births in the last year and children ever born for women aged 15 to 49 years were available for all four districts. Using these data and standard fertility schedules, adjusted age-specific fertility rates and total fertility rates for the two periods were computed using the Relational Gompertz model [20,21]. The Relational Gompertz model assumes that the standard fertility schedule used approximately represents the pattern of the fertility distribution in the population, changes in fertility have been relatively gradual and had a similar effect on all age groups, fertility of reported recent births is quite accurate and the parities reported by women aged 20–29 or 20–34 are also accurate [20]. From the adjusted age-specific fertility rates, annual rates of change of age-specific fertility rates between the 2002 and 2012 censuses were computed and used to estimate annual age-specific fertility rates for each year 2013 to 2016. Finally, annual estimates of number of births were obtained by multiplying estimates of mid-year population by the age-specific fertility rates. Mid-year population of women age 15 to 49 years was estimated using a combination of linear and geometric interpolation and extrapolation to years 2013–16 based on published district census data disaggregated by age and sex for 2002 and 2012 [12,22].

### Quantification of CRVS milestones

The quantification of milestones in the birth registration system was analysed and presented using Sankey charts, that show where births go missing in the system [23]. Sankey charts were developed for both centralized and decentralized systems, and, within each system, births occurring in health facilities and in the community. For centralized districts, notified births for 2012 to 2016 were obtained from district registrars of Kibaha and Dodoma. These data represent the total number of annual birth notification forms received from health facilities within the districts. The true number of births occurring in health facilities was estimated using regional percentages of deliveries in health facilities estimated by the 2015–16 DHS. Community births were calculated as the difference between health facility births and total estimated births. In centralized districts, 72% of births occur in facilities, and in decentralized districts, 71% of births occur in facilities.

Birth notification greater than 100% is possible in centralized districts and would occur where notified births are higher than the estimated number of births. This would be caused by an under-estimate of the true number of births occurring in health facilities due to either the estimate of the true number of all births or the percentage of all births occurring in health facilities (from the DHS estimates for the region) being too low. Another reason for birth notification exceeding 100% in Kibaha District could be due to its geographical nature, which makes it easily accessible by mothers residing outside the district tor better health care and whose births may be included as part of that district’s figures. Removing the numbers of births to mothers residing outside of Kibaha District from notification data was not possible.

## Results

### Completeness of birth registration/certification

**Figure 4.**
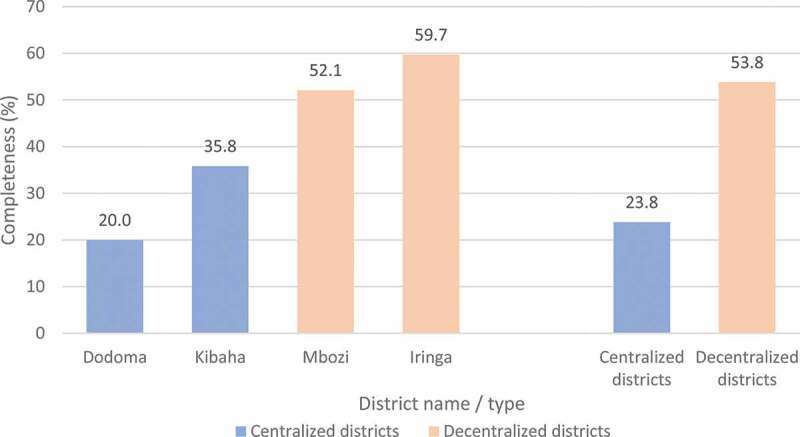
Completeness of birth registration/certification (%), births that occurred from 2012 to 2016 that were registered by early 2017*, by district and whether district decentralized during study period

For births that occurred during the period 2012 to 2016, the completeness of birth registration/certification in districts that decentralized during the study period (54%) was more than two times higher than districts that remained centralized during the study period (24%) (Figure 4). Completeness in decentralized districts was 60% in Iringa and 52% in Mbozi. In centralized districts, completeness was 36% in Kibaha and 20% in Dodoma.

*Centralized districts as of 6 January 2017; Decentralized districts as of 27 April 2017.

Trends over the period show that birth registration/certification completeness increased in districts that decentralized during the study period, reaching 60% in 2016 (Table 1). In centralized districts, completeness of births registered by early 2017 fell from 31% in 2014 to 14% in 2016, however this lower completeness was partly due to the earlier extraction date of data for this study of 6 January 2017. By the end of July 2019, there was completeness of 35.8% for 2015 and 2016 births in the centralized districts, which was higher than for the figures for 2012–14 for these districts reported in Table 1, but still lower than in decentralized districts.

**Table 1.** Completeness of birth registration/certification (%) by district, whether district decentralized during study period and year of birth, births registered by early 2017^a^

District	Year of Birth					2012–16
	2012	2013	2014	2015	2016	
**Centralized Districts**	**25.9**	**26.6**	**30.7**	**21.9**	**14.3**	**23.8**
Dodoma District	21.9	22.4	26.2	17.7	12.1	20.0
Kibaha District	38.5	40.1	45.0	35.1	21.2	35.8
**Decentralized Districts**	**48.4**	**54.1**	**53.5**	**52.6**	**60.0**	**53.8**
Mbozi District	49.1	54.9	51.9	49.6	54.7	52.1
Iringa Municipal	46.0	51.0	59.2	62.5	78.1	59.7

^a^Centralized districts as of 6 January 2017; Decentralized districts as of 27 April 2017.

The completeness of birth registration/certification, when only including births registered within 12 months of occurrence, increased sharply in the year of decentralization in Mbozi district in 2013 (from 15% in 2012 to 36% in 2013), before falling to 13% in 2015 and increasing again to 53% in 2016 (Table 2). In Iringa, there was also a sharp increase in the year of decentralization in 2016 from 1% in 2014 and 17% in 2015 to 67% in 2016. In districts that were centralized throughout the study period, completeness did not exhibit any clear increase.

**Table 2.** Completeness of birth registration/certification (%) by district and year of birth for births registered within 12 months from date of birth, 2012–16

District Name	Year of Birth					2012–16
	2012	2013	2014	2015	2016	
Dodoma District	14.5	16.6	21.4	15.4	12.1	16.0
Kibaha District	22.1	26.6	33.7	30.4	21.2	26.8
Mbozi District	14.8	35.7	19.0	12.6	53.3	27.2
Iringa Municipal	0.1	0.4	0.9	17.1	66.6	17.7

### Quantification of registration milestones

The steps of birth registration quantified were notification, validation, registration and certification. There is a time gap between registration and certification; all registered births are also certified. In both systems reported notified births are those which were validated and registered. It is not easy to quantify all notified births which are not validated and later registered, because once a notification tag is issued, it is dependent upon the registrar to check its validity; if it is not valid, it is discarded and the event is not registered.

Overall, during 2012 to 2016 the districts that remained centralized had a higher percentage of births (68%) notified compared with districts that decentralized (54%) (Table 3). In Kibaha, an estimated 101% of all births were notified during the period. As detailed in the Quantification of CRVS Milestones section, birth notification exceeds 100% in some years in centralized districts where notified births are higher than the estimated number of births. The centralized district of Dodoma experienced a decline in notification in 2015 and 2016 while Iringa, which decentralized in 2016, had an increase during the period; Kibaha and Mbozi did not have an obvious trend in notifications.

**Table 3.** Birth notification and registration/certification (%), whether district decentralized during study period, 2012–2016, births registered by early 2017^a^

Centralized	Estimated Births	Notified	Registered/ certified	Decentralized	Estimated Births	Notified	Registered/ certified
District/Year	Number	%	%	District/Year	Number	%	%
Total	113,654	67.5	23.8	Total	138,489	53.8	53.8
Dodoma (Total)	86,221	57.0	20.0	Mbozi (Total)	107,364	52.1	52.1
2012	16,782	52.7	21.9	2012	20,903	49.1	49.1
2013	17,009	59.9	22.4	2013	21,177	54.9	54.9
2014	17,240	79.9	26.2	2014	21,461	51.9	51.9
2015	17,475	54.6	17.7	2015	21,758	49.6	49.6
2016	17,715	38.3	12.1	2016	22,066	54.7	54.7
Kibaha (Total)	27,433	100.6	35.8	Iringa (Total)	31,125	59.7	59.7
2012	5,306	62.9	38.5	2012	5,960	46.0	46.0
2013	5,395	119.0	40.1	2013	6,089	51.0	51.0
2014	5,485	122.8	45.0	2014	6,221	59.2	59.2
2015	5,577	97.3	35.1	2015	6,357	62.5	62.5
2016	5,671	100.3	21.2	2016	6,498	78.1	78.1

^a^Centralized districts as of 6 January 2017; Decentralized districts as of 27 April 2017.

Despite districts that remained centralized having higher levels of notification of births, the districts that decentralized performed much better on certification due to notification, registration and certification all occurring at the same time (54% of all estimated births) compared with just over one-third of births in centralized districts (or 24% of all estimated births). In Kibaha in 2016, only one-fifth of notified births were certified.

**Figure 5.**
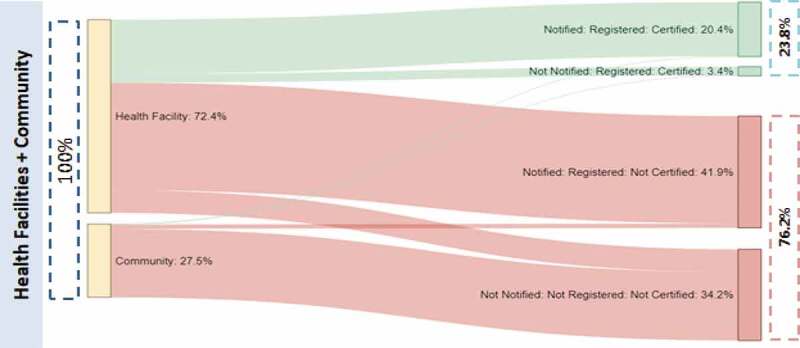
Sankey charts of flow of births by place of occurrence, districts that remained centralized during the study period, 2012–2016

The performance of birth registration for births that occur in health facilities and the community were analysed separately using Sankey diagrams showing flows of notified and non-notified births towards registration and certification. While notification happens in health facilities, any birth can be notified within 90 days when the parent gets into contact with a health facility. Analysis from the Sankey charts for districts that remained centralized shows that births that occurred in health facilities were more likely to be notified, registered and lastly certified than births that occurred in the community; about 84% of health facility births were notified as compared to only 6% of community births. One-third of all births that occurred in health facilities in centralized districts were certified compared with less than 1% of community births (Figure 5). The vast majority of certified births were initially notified (20% out of 24%) and were almost all from health facilities.

**Figure 6.**
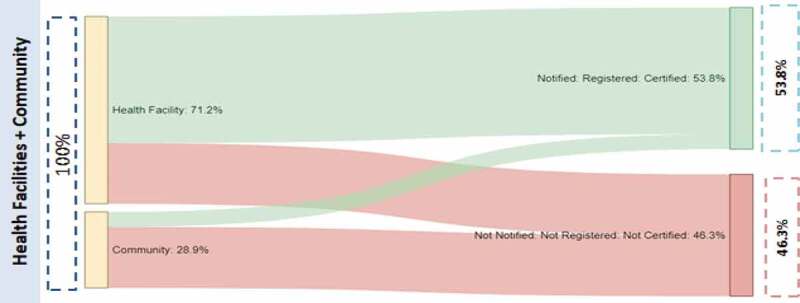
Sankey charts of flow of births by place of occurrence, districts that decentralized during the study period, 2012–2016

In districts that decentralized during the study period, notification, registration and certification occur together, with births needing to be notified within 5 years of occurrence. Similar results were observed under this system, with health facility births more likely to be notified, registered and finally certified (68%) as compared to community births (20%), although the figure for community births is higher than for centralized districts (Figure 6). All certified births passed through the notification step, with a significant portion of community births getting notified, registered and finally certified (Figure 6) as compared to the centralized system (Figure 5).

68% of health facility births and 20% of community births are notified, registered and certified.

## Discussion

Findings from the study show that in two districts in Tanzania where a decentralized birth registration system was introduced, there was a substantial increase in completeness of birth registration and certification within 12 months compared with districts that retained a centralized system. For births occurring in 2016, 60% of births in decentralized districts were registered (78% in Iringa, 55% in Mbozi) compared with just 14% in centralized districts and the estimated 24% completeness for all Tanzania. The introduction of the decentralized system had a significant impact in Iringa in particular, with completeness of registration and certification within 12 months increasing from less than 1% in 2014 to 67% in 2016. There was also an increase in completeness in Mbozi in 2013, before falling to 2015 and increasing again in 2016, three years after the introduction of decentralisation, because RITA, with support from UNICEF, re-trained registration assistants, public awareness campaigns were conducted and improved devices for data uploading were introduced.

The introduction of the decentralized system also resulted in a higher level of registration and certification of births that occurred between 2012 and 2016 when compared with the centralized system, because all births from 2012 were eligible to be registered and certified under the new system. Closer examination of the registration milestones shows that a higher percentage of births in centralized districts were notified than in decentralized districts. However, only about one-third of these notified births were registered by early 2017, compared with all births in the decentralized system. These findings are similar to those by Kabadi et al that showed a routine CRVS system certified very few births following notification [24]. The findings also of much interest because we were unable to find any empirical data from previous studies of any association between decentralisation and birth registration.

The introduction of the U5BRI in Iringa and Mbozi has improved access to birth registration by increasing the numbers of registration centres from only two district offices to 140 centres comprising of wards and health facilities. In both districts, the majority of parents can now access the service at no cost within five kilometers from their residence regardless of their rural/urban setting. This finding is consistent with evidence from a study that identified transport and cost as among issues that hinder households to certify births [24]. Further, in this decentralized system the steps in birth registration have been merged; that is notification, validation, registration and certification happen together. This significantly improved the registration and certification steps, as demonstrated by the low percentage of notified births in the centralized system that are eventual registered/certified. The merging of steps also has significantly improved the timeliness of birth registration. A delay of several years between the registration of births and deaths and publication of the corresponding statistics will mean that the information will be of only limited use in guiding programme planning and implementation [3].

Although the introduction of the decentralized birth registration system has improved birth registration, completeness is still well below 100%. Eighty per cent of community births and 32% of facility births are not notified; without improvement in notification of births, birth registration will remain incomplete. Another issue preventing the greater completeness in decentralized districts is the increased time to notify a birth from 90 days in the centralized system to five years. Parents may simply delay registering a child since they have ample time and in some cases they may forget or fail to register the child within defined period.

This study showed substantial increases in birth registration completeness in two districts during the year that each decentralized the registration process. However, the evidence is less clear about the longer-term sustainability of the decentralized system, which is a limitation of the study. In Mbozi, there was a decline in completeness in 2014 and 2015 before another increase in 2016 due to the interventions described above, while in Iringa we do not have any data after the year of decentralization. It is apparent that although the introduction of a decentralized system, based on this evidence, may lead to an immediate increase in completeness, continued efforts need to occur to sustain these improvements over the medium- and long-term. Another limitation of the study is that the estimates of birth registration completeness are reliant on estimating total annual births in each district using demographic methods that make assumptions of population dynamics. The higher number of notifications in Kibaha than estimated annual births suggests that either the estimate of annual births is too low, or potentially births of non-residents of this district are included. However, the impact of such issues would most likely be consistent over the period of analysis, and so the trends in completeness would be unaffected.

The study provides some evidence on the immediate impact of the new decentralized birth registration system on completeness, but also, based on the experience of Mbozi, demonstrates that continued efforts are necessary to sustain these improvements. This lesson is important for other districts in Tanzania that will introduce this decentralized system. Additional qualitative research could further inform how to improve birth registration amongst sections of the population for whom it remains low, even in decentralized districts, and which might highlight how to raise awareness of the benefits of birth registration. The study findings also suggest that one strategy for countries to attain complete birth registration by 2030, as stated in Sustainable Development Goal 16.9, is to consider implementation of decentralized district-level registration systems and processes that include increased numbers of registration points and more efficient steps in the registration and certification process. Further studies could be extended to determine how such decentralization also benefits the completeness of death registration which is currently lower than birth registration.

## Conclusion

This study has shown that, compared with the two districts that retained a centralized birth registration system, completeness of birth registration by the end of the study period was higher in the two districts that decentralized their system and that completeness within 12 months of birth increased substantially during the year they decentralized. However, the experience in one of the decentralized districts suggests that ongoing efforts are required to maintain those initial gains to later years, and more information should be collected from other decentralized districts to identify best practices that can inform roll-out of decentralized birth registration to other districts. The steps in birth registration have varying quantities between the two systems; while a centralized system indicates better performance in notification but poorer in registration, a decentralized system has been able to improve both notification and registration steps but after varying notification times. It can be concluded that decentralization is important towards reaching completeness of birth registration, however, the need to re-consider the reduction of notification times under this system is important considering timeliness of production of vital statistics that can be used by decision-makers. Improvement of birth registration completeness will provide more Tanzanians with legal identity, improve their access to social security benefits and health and education services, and also strengthen fertility statistics.

## Data Availability

Data used were notification data from the district registrars of Kibaha and Dodoma and registration and certification data of the Registration Insolvency and Trusteeship Agency (RITA). These datasets are not available for public use, however aggregated data from the study are available from Christopher Sanga by reasonable request.

## Paper context

In Tanzania only one-quarter of births are registered. A pilot decentralized birth registration system sought to increase registration completeness by introducing more registration points (e.g. local ward level offices and health facilities) and merging notification, registration and certification steps. The decentralized system resulted in significantly higher completeness of registration and certification than in centralized districts where no such changes occurred. The study provides information for other districts in Tanzania that will introduce this decentralized system to improve birth registration.
